# A multivariate approach to identify association between peripheral blood DNA methylation and cerebrospinal fluid biomarkers of Alzheimer disease

**DOI:** 10.1038/s41598-025-22004-3

**Published:** 2025-11-03

**Authors:** Bowei Xiao, Yixiao Zeng, Kathleen Oros Klein, Bianca Granato, Mathieu Blanchette, Xiaojian Shao, Celia M. T. Greenwood

**Affiliations:** 1 Quantitative Life Sciences, McGill University, Montreal, QC Canada; 2 Lady Davis Institute for Medical Research, Jewish General Hospital, Montreal, QC Canada; 3 School of Computer Science, McGill University, Montreal, QC Canada; 4 Digital Technologies Research Centre, National Research Council Canada, Ottawa, ON Canada; 5 Ottawa Institute of Systems Biology, Department of Biochemistry Microbiology and Immunology, University of Ottawa, Ottawa, ON Canada; 6 Gerald Bronfman Department of Oncology, McGill University, Montreal, QC Canada; 7 Department of Epidemiology, Biostatistics and Occupational Health, McGill University, Montreal, QC Canada

**Keywords:** Alzheimer disease, DNA methylation, Cerebrospinal fluid biomarkers, multivariate analysis, penalized models, dimension reduction, CLSA, DNA methylation, Statistical methods, Alzheimer's disease, Biomarkers

## Abstract

DNA methylation has been shown to play a crucial role in many diseases, including Alzheimer’s disease (AD). Although many studies have correlated DNA methylation in blood samples with risk of clinical AD diagnosis, few have examined links with AD neuropathology. Using data from the Alzheimer’s Disease Neuroimaging Initiative (ADNI) study, we investigate the associations between peripheral blood DNA methylation and three AD-associated biomarkers in cerebrospinal fluid: amyloid-$\beta$, phosphorylated tau-181, and total tau using an innovative multivariate approach. In our approach, we first adjusted the methylation values for covariates that have known wide-spread effects on methylation. We then developed and implemented a multivariate penalized model to find associations, jointly, between CSF biomarkers and sets of methylation residuals defined by regions around each gene. These penalized models then selected probes showing associations with one or more CSF biomarkers. We demonstrate, using both simulations and actual data, that our proposed multivariate approach is beneficial for detecting weak signals. We also provide complementary validation using data from the Canadian Longitudinal Study on Aging. Our multivariate strategy has the potential to increase feature selection accuracy among correlated predictors in epigenetic studies.

## Introduction

Alzheimer’s disease (AD) is the most common type of dementia and is a significant public health concern, contributing to an enormous global social and financial burden. It is an aging-associated neurodegenerative disorder characterized by progressive cognitive decline and memory loss^1,2^. AD is characterized by abnormal accumulation of amyloid plaques, hyperphosphorylated tau tangles, synaptic dysfunction, and eventually neuron cell death^3–5^. Current treatments for AD are limited and do not offer a cure. In addition, there are no effective strategies available for early detection, which might allow interventions to slow disease progression. One reason for this gap is the poor understanding of molecular mechanisms involved in disease progression and how epidemiologic risk factors relate to this deterioration.

Aging is well known to be the strongest risk factor for AD^6,7^. Furthermore, both genetic and lifestyle factors play a role in determining individual risk^8^. The largest known genetic association is with *APOE4*, where the presence of two copies of the apolipoprotein $\epsilon$4 allele is known to substantially increase the risk of AD^9,10^. Non-genetic factors, such as environmental and lifestyle factors, also contribute importantly to AD risk^11,12^.

DNA methylation, the addition of a methyl molecule to a cytosine, is a common and important epigenetic mechanism. It is known to change with age, exposure to environmental factors and disease states, and has been studied as a promising biomarker to understand and assess AD^13^. DNA methylation’s relationship with age is so reliable that it has been used to estimate chronological age, thereby defining the concept of epigenetic age acceleration, which is the difference between the estimated age based on DNA methylation, known as the epigenetic age, and chronological age^14^. Epigenetic age acceleration has been linked to neuropathological measurements, such as amyloid load or neuritic plaques in the brains of individuals with AD^15–17^.

Epigenome-wide DNA methylation studies (EWAS) have characterized DNA methylation alterations for AD in tissues across various postmortem brain regions^18–20^. Progression of AD, as measured by Braak stages, has also shown association with DNA methylation differences across different regions of postmortem brains, specifically at *ANK1*, *RHBDF2*, and *BIN2*^1,21^. Furthermore, EWAS in peripheral blood have shown differences between AD patients and controls^22–25^. Studying peripheral blood to understand a disease of the brain can be useful, since changes in peripheral blood DNA methylation have been found for several traits that primarily affect other tissues^26,27^. Specifically, the EWAS from Vasanthakumar et al.^25^ was performed using DNA methylation measured in peripheral blood samples from 653 participants in the Alzheimer’s Disease Neuroimaging Initiative (ADNI) consortium. ADNI was launched in 2003 to provide resources to help improve understanding of AD^28^. They identified several loci showing differential methylation between individuals with AD, mild cognitive impairment (MCI), and age-matched healthy control (CN) groups^25^.

Cerebrospinal fluid (CSF) biomarkers, specifically amyloid-$\beta _{42}$ (A$\beta$42) proteins, phosphorylated tau 181 protein (p-tau), total tau protein (t-tau), referred to here, respectively, as (A/T/N), are known to show association with AD and, moreover, to distinguish subtypes of AD through what has been termed the A/T/N framework^29,30^. Specific values and combinations of A/T/N levels have been proposed to offer high precision for the early diagnosis of AD, and have been used as research criteria to diagnose AD^31–33^. Instead of searching for associations between DNA methylation and the clinical phenotype of AD, some researchers have explored the relationships between peripheral blood DNA methylation and these CSF biomarker levels. For example, an EWAS analysis was conducted on a subset of the ADNI cohort (i.e. 123 CN and 79 AD) to search for associations between DNA methylation and CSF (A/T/N)^34^. This study identified loci that were associated with A, T, and N in either the CN or AD groups, but the associated loci overlapped little between the two groups. Another study investigated the potential for predicting CSF A/T/N biomarkers using epigenetic age acceleration estimates^35^, although their results had poor predictive performance. We hypothesize that there may be other DNA methylation signatures that contribute to CSF A/T/N biomarkers in ways that are not related to aging.

An important challenge that arises when examining associations between DNA methylation and AD diagnosis in ADNI data is that the diagnosis of AD may have occurred long before the start of the study. Participants were initially recruited based on their diagnosis (i.e. AD, MCI or CN), and were reassessed at subsequent yearly visits. For participants recruited with an pre-existing AD diagnosis, the original date of diagnosis tends to be imprecise or unknown. This unobserved period of time will impact the interpretability of any associations, since the brain deteriorates after diagnosis^36,37^. The CSF biomarkers, on the other hand, are not only good candidates to proxy AD disease status and severity^29^, but in ADNI they were also measured at nearly the same time as the DNA methylation data were collected.

Existing studies of DNA methylation, A/T/N, and AD have either focused on univariate associations (analyzing one probe and one phenotype at a time) or have built models to predict one CSF biomarker at a time. That is, previous work has rarely accommodated correlations between CpG sites or explored associations with the three correlated biomarkers A, T, and N. In this work, we developed and fit novel multivariate penalized models to estimate associations between the CSF biomarkers, jointly, and methylation levels at sets of probes defined by gene positions. We chose to build penalized models to reliably estimate which of the methylation probes in or near a gene region are associated with the CSF biomarker levels. This new method and innovative strategy leads to new insights into gene regulation of CSF biomarkers and AD.

## Results

After applying quality control filters, data normalization and various selection criteria, we analyzed $n=540$ ADNI participants where DNA methylation data and (A/T/N) CSF biomarker data were available, including 88 AD cases, 278 MCI, and 174 controls (CN). The phenotypic data used for our analyses was taken from the visit that was closest in time to when the blood was drawn for methylation analysis (mean difference of 3 days). Details on selection of participants and our quality control criteria are in the “Methods” section. Descriptive statistics of demographic variables and CSF biomarkers for the participants in this study can be seen in Table 1.

**Fig. 1:**
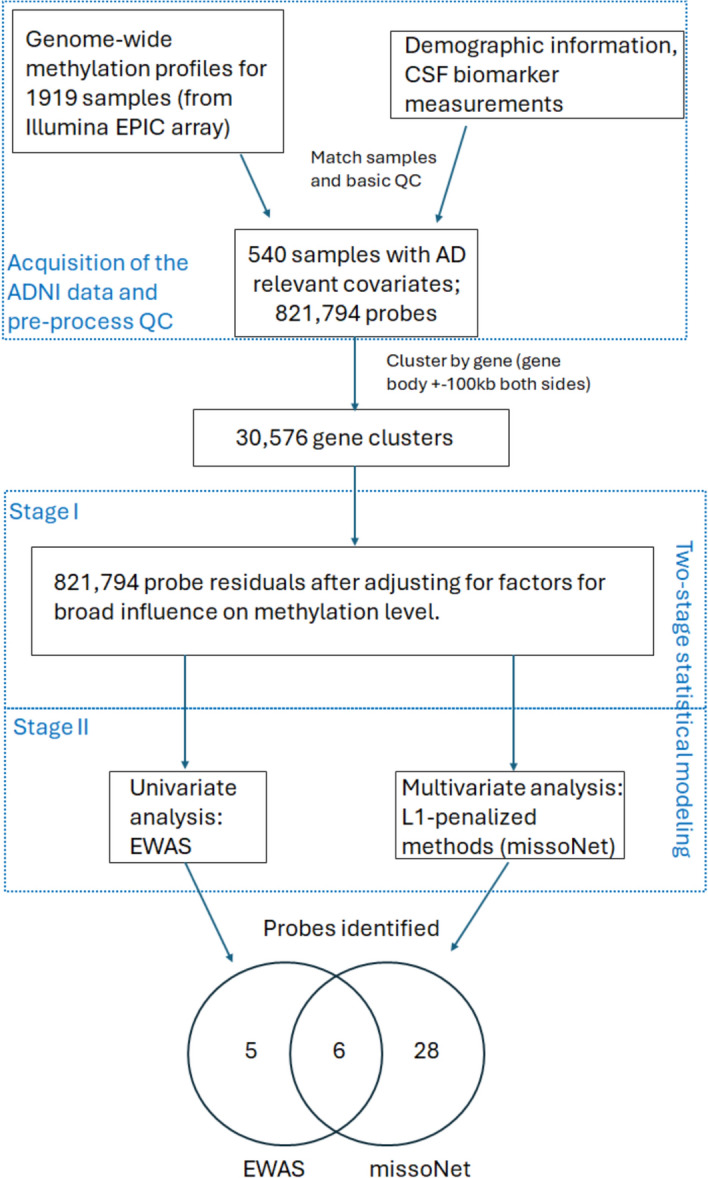
Overview of our strategy for analyzing associations between CSF biomarkers and DNA methylation in ADNI data. Details of each step can be found in the corresponding sections in the “Methods”.

Our overall workflow is highlighted in Fig. 1 and full details are provided in the “Methods” section. Since our goal was to implement a multivariate analysis of the CSF biomarkers, we started by examining correlations among the three A/T/N measures (We will name these raw measurements as $\hbox {A}^{*}/\hbox {T}^{*}/\hbox {N}^{*}$). There is a high correlation between $\hbox {T}^*$ and $\hbox {N}^*$ (correlation=0.981). In order to avoid problems associated with collinearity and skewness, we log-transformed and scaled these raw measurements, and then calculated the mean (M) and difference (D) of $\hbox {N}^*$ and $\hbox {T}^*$. We will refer to the log-transformed and scaled $\hbox {A}^*$ as A. Henceforth, our multivariate analyses used the scaled, transformed A, M, and D as phenotypes (see “Methods”). Note that we continue to refer to the transformed phenotypes as ‘CSF biomarkers’.

Supplementary Figs. 1 and 2 display distributions of correlations between methylation probes and the CSF biomarkers, both raw $\hbox {A}^*/\hbox {T}^*/\hbox {N}^*$ counts and transformed *A*/*M*/*D*, giving a genome-wide perspective. Most of the probes in the genome have minimal correlation with the CSF biomarkers. However, there are some probes with correlations as large as ± 0.3 for transformed CSF biomarkers. We also examined how the CSF biomarkers were associated with the variability of the methylation data by looking at the correlation between the biomarkers and the first principal component (PC) of the methylation data, which accounts for 18.4% of the total variation. Most transformed biomarkers are negatively associated with the first PC, although correlations are weak (A: −0.046, M: −0.065, D: 0.022).

**Table 1:** Characteristics of the ADNI participants who are included in this study.

	Cases (AD)	Controls (CN+MCI)	CN only	MCI only
Participants	88	452	174	278
Age at visit: mean (s.d.)	75.77 (8.12)	74.33 (7.42)	76.33 (6.57)	73.07 (7.65)
% Female (count)	39.8 (35)	46.7 (211)	50.6 (88)	44.2 (123)
Average years of education (s.d.)	15.99 (2.77)	16.28 (2.64)	16.33 (2.69)	16.24 (2.61)
$\ge$ 1 *APOE4* allele (%)	73.9	34.7	24.7	41.0
$\hbox {A}^*$ median (IQR)	609.70 (500.75, 800.80)	1048.00 (697.90, 1625.25)	1276.00 (850.55, 1705.00)	922.50 (663.00, 1432.50)
$\hbox {T}^*$ median (IQR)	34.23 (25.64, 44.49)	22.11 (16.64, 30.39)	21.05 (16.24, 28.13)	23.33 (17.03, 32.41)
$\hbox {N}^*$ median (IQR)	340.65 (280.05, 448.98)	245.15 (187.85, 320.50)	234.60 (186.65, 304.15)	253.30 (190.95, 327.60)
A median (IQR)	−0.86 (−1.27, −0.30)	0.26 (−0.58,1.16)	0.66 (−0.17,1.26)	−0.007 (−0.68,0.90)
M median (IQR)	0.73 (0.11,1.26)	−0.18 (−0.79, 0.45)	−0.30 (−0.84,0.33)	−0.12 (−0.77,0.55)
D median (IQR)	−0.03 (−0.14,0.11)	0.0044 (−0.12,0.12)	0.028 (−0.086, 0.13)	−0.013 (−0.14,0.12)

AD: Alzheimer disease. CN: Cognitively normal. MCI: Mild cognitive impairment. IQR: Inter-quartile range. $\hbox {A}^*$: amyloid-$\beta$ 42. $\hbox {T}^*$: 181-phosphorylated tau. $\hbox {N}^*$: total tau. A: scaled (log $\hbox {A}^*$), M: (scaled (log $\hbox {T}^*$) + scaled(log $\hbox {N}^*$))/2. D: scaled (log $\hbox {N}^*$) - scaled (log $\hbox {T}^*$). Scaling does both centering and scaling variance to 1.

### Epigenome-wide association analyses

We undertook a two-stage strategy to run genome-wide association analyses between DNA methylation and CSF biomarkers. In the first stage, we used linear models to test association between the logit of the methylation levels at each probe, and a set of covariates that are well-known to influence DNA methylation levels broadly across the genome, namely age, sex, and immune cell type composition, as well as years of education (as a proxy for socioeconomic status). We also included epigenetic age acceleration, the difference between chronological age and the estimated epigenetic age (see “Methods”). After running these linear models for all probes separately, we calculated the residuals of the fitted models for use in the next stage of our analyses. This strategy allowed our second stage to focus on the relationship between specific probes’ DNA methylation levels and the CSF biomarker values. In the second stage, we tested the pairwise associations between transformed CSF biomarkers and the methylation residuals from stage 1, adjusting for diagnosis of AD and the *APOE4* allele counts, both of which are potential confounders of the CSF biomarker-methylation associations. As mentioned in the Introduction, due to the timing of AD diagnosis in ADNI, methylation cannot be treated as a mediator between CSF biomarkers and AD diagnosis.

**Fig. 2:**
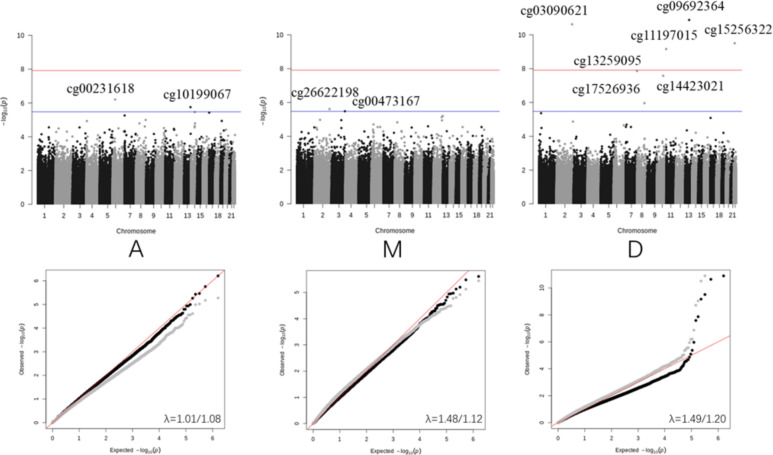
EWAS results shown with Manhattan plots (upper row) and Q-Q plots (lower row) for the three biomarker phenotypes A, M and D. In the Manhattan plots, two horizontal lines indicate the genome-wide significance level (red) and the suggestive significance level (blue). The grey dots in the QQ-plot show the *p* values before the bacon correction and the black dots show the *p* values after the bacon correction. The line of $y=x$ is shown in red. The inflation factors $\lambda$ before the bacon correction are (left to right) 1.01, 1.48, and 1.49, and after correction, 1.08, 1.12, and 1.20.

We analyzed 776,848 CpGs independently in our EWAS for each of the three CSF biomarker phenotypes (A, M, D). Upon obtaining the test statistics for each probe and each phenotype, we applied the *bacon* correction^38^ to correct the distribution of *p* values for bias and inflation (Fig. 2). After the bacon corrections, 4 probes (cg03090621, cg11197015, cg09692364, cg15256322) were identified as genome-wide significant for their association with D, based on a threshold of $\alpha =\frac{3.6e-8}{3}$ after correcting for multiple testing^39^. There are 7 additional probes identified with a suggestive level of statistical significance^39^ after correcting for multiple testing ($\alpha =\frac{1e-5}{3}$). The lists of probes selected by EWAS before and after applying the bacon correction can be found in Supplementary Tables 1 and 2.

### Joint analyses of three biomarker phenotypes and multiple probes

While the probes identified by our EWAS may provide some understanding of the neuropathology of AD, we suspect that there are additional factors that were not captured. In particular, the paucity of associated probes may be due to a lack of statistical power. While the two-stage model we proposed above adjusts for several covariates known to influence AD risk, the EWAS analysis cannot benefit from correlations that may be present among the CSF biomarkers. It also does not leverage correlations in methylation values among neighbouring probes, even though methylation levels at nearby positions can be tightly correlated. Therefore, we hypothesized that a joint analysis, using three CSF measures and multiple probes simultaneously, may achieve greater power to detect associations of interest. We clustered neighboring probes based on their location with respect to annotated genes. For each gene G, a group of probes was created including probes located in the body of G or the 100 kb flanking regions. Overall, we created 30,576 gene clusters for downstream analysis. Note that probes can be assigned to multiple clusters. We will refer to these sets as gene clusters, or simply ‘clusters’ throughout this manuscript. The distribution of the number of probes in each gene cluster is shown in Supplementary Fig. 3; the median number of probes per cluster was 114 probes, and 93.53% of the gene cluster sizes contain at most 300 probes. For computational efficiency reasons, clusters larger than 300 probes were subdivided into smaller clusters of size at most 300 (see “Methods”). We used *missoNet*^40^, a recently developed L1-penalized multivariate method (manuscript in preparation), to analyse each gene cluster and identify probes associated with one or more of the CSF biomarkers. *missoNet* can simultaneously account for correlations among probes as well as among multiple phenotypes. It supports parameter tuning based on various information criteria (e.g. Bayesian Information Criteria; BIC) or on k-fold cross-validation (CV). Information criteria favour model parsimony and are asymptotically consistent, whereas CV directly estimates out-of-sample predictive error – each induces a different sensitivity–specificity trade-off.

**Fig. 3:**
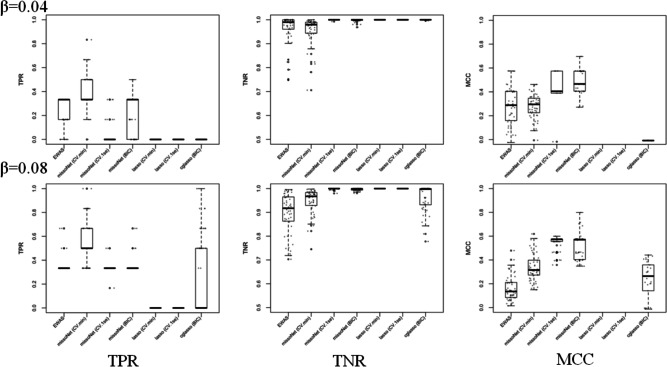
Simulation performance across 50 simulated datasets between different multivariate methods with two different true effect sizes (top row effect size = 0.04, bottom row effect size = 0.08). Under each scenario, we compared 7 different combinations of L1-penalization methods and selection criteria. We evaluated the performances of each methods using True positive rate (TPR), True negative rate (TNR) and Matthews correlation coefficient (MCC). Overall, *missoNet*-BIC performed the best among other methods. The results for each simulated dataset (grey dots) are overlaid with boxplots.

Since the optimal penalty depends on sample size, signal sparsity, and noise level, we conducted a simulation study, varying these factors, to determine whether BIC or CV achieved the best variable selection on data resembling ours. Our simulation study sought to evaluate the performance of our proposed analytic pipeline and to assess the merit of our hypothesis of increased power. The simulation was designed to replicate the types of patterns observed in the actual datasets, ensuring that the synthetic data accurately reflected the natural variability and correlation observed in the ADNI data. We built the simulations using ADNI methylation data of randomly selected gene clusters, and then simulated the three phenotypes. To model the correlation structure for the three responses (A, M, and D), an empirical variance-covariance matrix derived from the real data was used. We simulated a null scenario where no probes are associated with any phenotype, and additional scenarios where true association exists under different setups. We evaluated the performance of each method based on the balance between sensitivity and specificity of the probe selection task, measured by Matthews correlation coefficient (MCC)^41^. In our simulation comparison, we have also included another multivariate penalized method, *cglasso*^42^. Complete information on the simulation settings and results can be found in “Methods”, Fig. 3, Supplementary Section 2, and Supplementary Figs. 4 and 5. Our simulation results show that *missoNet* with BIC performs the best, in several different simulation settings, when compared with conventional EWAS or compared to other optimization methods, and we chose this approach for analysis of the ADNI data.

Returning to the ADNI data, we analyzed the association between each of these clusters and the CSF biomarkers with our penalized multivariate method, *missoNet*. The computational complexity and memory requirements of of *missoNet* are low and the entire analysis of the 30,576 clusters took less than 100 hours on a single CPU; see Supplementary Table 10 for details. For each gene cluster, we estimated coefficients for the associations between all probes and the biomarkers. By the nature of the model, most of the coefficients are penalized to zero. *missoNet* selected 34 probes as having non-zero effects for at least one CSF phenotype (Table 2). Since the methylation residuals were standardized prior to undertaking these analyses, each coefficient can be interpreted as an effect size, e.g. the predicted change in the transformed *A*, *M* or *D* for a one standard deviation change in the methylation residual. Table 2 also shows the probe locations relative to their respective genes, and among these 34 probes, 11 (or 32.4%) of them lie in or near the promoter or the first exon, suggesting possible regulatory roles. We have also examined the same associations using another multivariate approach, *cglasso*. Results from *cglasso* can be found in Supplementary Table 4, which identified more associated probes overall, but none with phenotype D. Table 2 also indicates the 10 probes (1 for *A*, 5 for *M* and 4 for *D*) that were identified by both *missoNet*-BIC and *cglasso*-BIC.

**Table 2:** Probes selected by *missoNet*.

Probe ID	Effect size ($\hat{\gamma }$) for			Annotated gene	Location in the gene
	A	M	D		
$\hbox {cg}14294953^+$	0	0.0746	0	NA	NA
$\hbox {cg}16027546^+$	0	0.0705	0	*OR10Q1*	1stExon; 5’UTR
$\hbox {cg}07079560^+$	0	0.0694	0	NA	NA
$\hbox {cg}08295410^+$	0	0.0642	0	*ADAM19*	Gene Body
$\hbox {cg}06570674^+$	-0.0590	0	0	NA	NA
$\hbox {cg}09692364^{*+}$	0	0	−0.0437	*TBC1D4*	Gene Body
$\hbox {cg}15256322^{*+}$	0	0	−0.0393	*OSBP2*	Gene Body
$\hbox {cg}03043160^+$	0	−0.0378	0	*TBC1D4*	TSS200
$\hbox {cg}03090621^*$	0	0	−0.0367	*EPHA4*	NA
$\hbox {cg}14423021^*$	0	0	−0.0291	*LINC00865*	Gene Body
$\hbox {cg}11197015^*$	0	0	−0.0264	*BNIP3*	TSS1500
cg06964350	0	0	−0.0177	*ZHX3/PLCG1*	3’UTR
$\hbox {cg}06732496^+$	0	0	0.0177	*ZMYND15/CXCL16*	TSS200
cg14432650	0	0	−0.0175	*SRSF4*	1stExon; 5’UTR
$\hbox {cg}17526936^*$	0	0	−0.0169	*OXR1*	Gene Body
cg14833412	0	0	−0.0160	NA	NA
cg16165404	0	0	−0.0158	*ANKRD36C*	Gene Body
cg23580662	0	0	−0.0157	*THSD7A*	Gene Body
$\hbox {cg}11525834^+$	0	0	0.0155	*ABHD11*	TSS200
cg09569166	0	0	−0.0153	*AKAP11*	5’UTR
cg02741177	0	0	−0.0143	*OPRPN*	TSS1500
cg14296561	0	0	0.0133	*BIRC3*	Gene Body
cg20974602	0	0	−0.0130	*ST6GAL1*	5’UTR; Gene Body
cg26853320	0	0	−0.0127	*PADI3*	Gene Body
cg15287044	0	0	−0.0123	*ZNF254*	TSS1500
cg03507018	0	0	−0.0123	*OR5H15*	TSS1500
cg24824725	0	0	−0.0123	*TCTN3*	TSS200
cg10842801	0	0	−0.0118	*HIVEP2*	5’UTR
cg09599862	0	0	0.0116	*SPOPL*	5’UTR
cg21136499	0	0	0.0116	*LFNG*	TSS200
cg26476169	0	0	0.011	*KCNB2*	Gene Body
cg08672314	0	0	0.011	NA	NA
cg25236398	0	5.791e−7	0	NA	NA
cg14755690	0	1.753e−7	0	*AHCYL2*	Gene Body

The estimated effect sizes, $\hat{\gamma }$, are for associations with the three transformed phenotypes A, M, and D. Their sign indicates whether higher methylation levels are associated with increases in the phenotype (positive) or decreases in the phenotype (negative). Probes were ranked in the table by their largest absolute effect size $| \hat{\gamma } |$. Of these probes, 27 of 34 were annotated to genes through the UCSC reference gene sets^43^. Of these 34 probes, the univariate EWAS analysis also identified 6 probes and these probes are indicated with an asterisk (*). The *cglasso* multivariate method identified 10 of these probes, and they are indicated with a plus sign (^+^). “NA” means that the manifest file did not annotate this probe to a gene.

### Replication study with data from CLSA

We undertook a replication of our findings in an independent dataset: the Canadian Longitudinal Study on Aging (CLSA) [Baseline Comprehensive Dataset version 6.0., Follow-up 1 Comprehensive Dataset version 5.0, and Epigenetics - Version 1.1 (s-1478)]^44^. Due to the design and goals of the CLSA study, even though AD-relevant phenotypes are measured, there are no data on CSF biomarkers nor direct measurement of the *APOE4* genotype. Therefore, for a complementary validation of our results, we tested for associations between the DNA methylation probes from Table 2 and two relevant measurements: self-reported AD status and REYII cognitive test score. These models were adjusted for covariates similar to those we used in stage 1 of our analysis of the ADNI data. While we did not find any associations at a Bonferroni-corrected threshold ($\alpha =0.05/(34*2)$), we found 2 probes showing association at $p<0.05$ with self-reported AD, and 6 probes showing association at $p<0.05$ with the REYII cognitive test; see Supplementary Section 1 and Supplementary Tables 5 and 6 for details.

## Discussion

In this study, we explored the relationships between DNA methylation in peripheral blood and CSF biomarkers known for their associations with AD. In simulations and in our analysis of ADNI data, we demonstrated the advantages of using an innovative multivariate approach that jointly models multiple phenotypes while accounting for correlations between variables. Based on our simulations, accounting for such correlations increased our power to detect associations while still controlling the false positive rate. Notably, this joint modelling strategy does not require consistency in the directions of the associations.

We have focused on analyzing the CSF biomarkers as our phenotypes of interest, while accounting for AD diagnosis status as a covariate in our model. We chose not to analyze the relationships between DNA methylation and AD diagnosis directly, since many of the AD cases had been diagnosed long in the past, prior to recruitment into ADNI. Not only is information on original diagnosis dates incomplete, but also AD leads to brain deterioration, which will then alter DNA methylation levels. In contrast, the CSF fluids were extracted closer in time to the DNA methylation measures. Since AD can lead to changes in both CSF biomarkers and DNA methylation levels, including the AD diagnosis in the model is necessary to avoid confounding of the biomarker-methylation associations. Furthermore, due to this ADNI recruitment design, we are not inducing collider bias by fitting models that are conditional on diagnosis. Our results must be interpreted as associations that remain after adjusting for AD status.

In our simulations, we compared our method, *missoNet*, with another multivariate penalization-based method, *cglasso*. There are several key differences between these two approaches. Firstly, the penalization formulations differ. *cglasso* includes an additional penalty term proportional to phenotype variances, resulting in stronger penalization when the training variances are smaller. Our analyses demonstrated that, due to this penalty structure, *cglasso* failed to identify any probes associated with the difference phenotype (D), likely because of its relatively lower variance. In contrast, the EWAS QQ-plot of results for D displays a clear departure from the null distribution for the smallest *p* values, adding credibility to the identified associations for these probes. Secondly, model selection methods also differ. *cglasso* relies exclusively on information criteria (e.g. AIC or BIC) for model selection, whereas *missoNet* supports both information criteria and a cross-validation-based approach, made possible by its computational efficiency. These differences in methodology and model selection strategies account for the discrepancies observed between the results reported in Supplementary Tables 3 and 4, and highlighted in Table 2.

Other authors have analyzed the methylation data in ADNI and its associations with AD (e.g. Vasanthakumar et al.^25^). Recently, we identified a related analysis of the methylation data in ADNI which also used the CSF biomarkers^34^. However, our approaches are fundamentally different: firstly, from the data ascertainment point of view, we included MCI samples and analyzed all ADNI participants at the same time, which substantially increased the sample size of our analysis. Secondly, our approaches focused on analyzing three transformed CSF biomarkers simultaneously, whereas Zhang et al.^34^ analyzed $\hbox {A}^*$, $\hbox {T}^*$ and $\hbox {N}^*$ separately. Unsurprisingly, the sets of probes we identified are not overlapping with theirs.

Although L1-penalized multivariate approaches have previously been applied to other problems, such as identifying sub-populations in single-cell data^45^, we have not yet seen these multivariate methods being applied to clinical epigenetics data. In ADNI, our multivariate approach successfully identified the signals detected by EWAS, but also highlighted additional loci. These new loci may help to better understand how DNA methylation is linked to CSF biomarker levels. We have explored the literature for these novel loci to gain insights into how or whether they are associated with CSF biomarkers or AD. Among our findings, some of these genes (e.g. *ST6GAL1*, *PLCG1*, *KCNB2*) have been associated with AD through genome-wide association studies^46–48^. Several other genes that we identified have also been found to be associated with AD in other contexts. For instance, in a TAU-mutant AD mouse model, *CXCL16* was upregulated at the late stage of AD progression in multiple brain areas, consistent with data obtained from human AD brains. This was further validated in human peripheral blood by comparing the expression level between AD patients and healthy controls^49^. As another example, expression of *OSBP2* was strongly and positively associated with AD progression in ADNI peripheral blood samples^50^; the probe we identified as associated with *D* (cg15256322) lies very close to the transcription start site of *OSBP2*. This probe has a negative effect on *D*, i.e. lower values of methylation residuals associate with higher values of *D*. Based on our definition of *D*, a higher value of *D* implies either less phosphorylated tau proteins or more total tau proteins. Hence, in our analysis, we can infer that dephosphorylation is associated with lower methylation, which thereby suggests upregulation of transcription at *OSBP2*^51^. This inference is consistent with the direction of the association found by Cohen et al.^50^.Furthermore, the ratio of phosphorylated tau and total tau is known to be associated with AD progression^52^.

There are certain limitations to our proposed two-stage modelling. Since we first adjusted DNA methylation levels for factors with wide-spread influence on DNA methylation (e.g. epigenetic age acceleration, sex, etc.), we are unable to explore whether these variables might modify the gene-based CSF-methylation associations. Since most study participants with AD were recruited after their diagnosis, it is not possible to use ADNI data to investigate whether methylation may mediate an association between, say, education and AD, nor between CSF biomarkers and AD. Furthermore, our multivariate penalized model does not allow inclusion of interaction terms, and therefore we may miss gene-specific effect modifications of the CSF biomarker associations with covariates such as age, sex and education, *APOE4* or AD status. However, we did fit univariate models including interactions with each of the latter two variables for the 34 methylation probes retained by our multivariate method, thereby performing a conditional search for effect modification (detailed in Supplementary Section 3 and full results can be found in Supplementary Tables 7-9). We found 5 of these identified probes were involved in a significant interaction term (threshold $\alpha =0.05/(34*3*2)$) with *APOE4* when analyzing the D biomarker. This suggests that there may be modifier effects of the *APOE4* genotype that are worth future study. We did not identify any interactions with the AD diagnosis.

In conclusion, we have proposed a novel multivariate approach to analyze DNA methylation data with multiple correlated phenotypes. We have shown through our simulations that, compared to traditional EWAS that ignores the correlations between and among CpG sites and CSF biomarkers, our multivariate approach is capable of detecting weaker signals and has better variable selection accuracy. In our analysis of ADNI data, we found strong and novel associations with our phenotype defined as the log difference between the total tau protein and phosphorylated tau-181. We were able to provide complementary validation of some of these probes in CLSA, by finding associations with two cognitive phenotypes. In an era where multi-omics approaches are becoming the norm, we foresee that multivariate approaches such as ours will enable discoveries that would otherwise be missed by univariate methods.

## Methods

Data preparation and analysis steps are described in detail below. Figure 1 summarizes the steps undertaken.

### Overview of ADNI participants

The ADNI portal^53^ provides access to genetic, epigenetic, and phenotypic data on their participants. We requested and obtained both DNA methylation measurements and participants’ diagnoses of AD and other demographic information that are relevant to AD.

### DNA Methylation data, and quality control steps (QC)

DNA methylation data derived from the Illumina Infinium MethylationEPIC Beadchip^54^ were obtained from the ADNI portal. This array provides a high-throughput DNA methylation profiling with a particular emphasis on capturing methylation at regulatory regions such as enhancers^55^. The data set contains DNA methylation data on 1919 samples from participants in three different phases of the study. The *minfi* package (Version 1.48.0)^56^ in R (cran.r-project.org; R version 4.2.1 for all analyses) was used to initially load and process the DNA methylation data. Then, we performed several basic quality control steps (QC). Briefly, we examined the detection *p* values for each of the methylation probes, and removed those with an average detection *p* value greater than 0.05 calculated across all samples. We further removed probes that had detection *p* values greater than 0.05 on half or more of our samples. The remaining probes were then normalized using the *preprocessFunnorm* function from the *minfi* package to minimize batch effects^57,58^. Probes that contain SNPs, as annotated by the Illumina EPIC array manifest file, were removed, as were cross-reactive probes^55,59^. We then further excluded probes with a detection *p* value $\ge 0.01$ on more than 20% of our samples to obtain only high-quality methylation data. After these steps, we retained 821,794 probes. None of the 1919 samples showed evidence of poor quality methylation data, so all were kept.

### AD phenotypes and related demographic information

We matched these methylation data to the phenotypic and demographic information provided through the ADNI portal. In cases where an individual had their DNA methylation assessed more than once, a single DNA methylation sample was retained, chosen to minimise the time between the dates of the DNA methylation blood extraction, and the phenotypic assessments, which led to 649 unique individuals being selected. Among these individuals, 540 participants had consented for collection of their CSF biomarker protein measurements, i.e. A/T/N. These proteins were measured using the multiplex xMAP Luminex platform (Luminex Corp, Austin, TX) with Innogenetics (INNO-BIA AlzBio3; Ghent, Belgium; for research use–only reagents) immunoassay kit–based reagents, and data were then normalized by the ADNI consortium^60^. High levels of these three protein biomarkers are known to demonstrate high sensitivity for AD disease; these data have been widely used clinically to create an optimal biomarker to distinguish AD patients from healthy controls^29,33^. For each individual enrolled in this study, the date of their annual visit to the clinic was recorded, along with their chronological age and AD diagnosis at that visit (either AD, mild cognitive impairment (MCI), or normal control (CN)). We also gathered their reported sex, years of education (as a proxy for socioeconomic status), and their *APOE4* allele counts^61^ from the ADNI portal.

Since DNA methylation levels vary significantly by cell type, we estimated the proportions of the common cell types occurring in blood from the DNA methylation data. Specifically, proportions of B cells, NK cells, CD4T cells, CD8T cells, monocytes, neutrophils, and eosinophils were estimated in each sample using the R package *EpiDISH* (Version 2.18.0)^62^. Then, these proportions were dimension-reduced using principal component analysis (PCA), and the first three components, which capture more than 95% of the variance, were included in subsequent analyses. Lastly, we estimated epigenetic age from the DNA methylation probes extracted from the peripheral blood using the method proposed by Horvath^14^, and then calculated epigenetic age acceleration (EAA), the difference between epigenetic age and chronological age. This difference can capture general epigenetic trends in aging that are usually not captured by the general trends with age. In our models, we adjusted for both age and EAA.

In the ADNI data, the raw A/T/N measurements ($\hbox {A}^*$, $\hbox {T}^*$, $\hbox {N}^*$) are skewed, and have very different scales, which can make interpreting estimated effect sizes challenging . Furthermore, there is a high correlation between $\hbox {T}^*$ and $\hbox {N}^*$ (correlation$=0.981$ and the conditional correlation between $\hbox {T}^*$ and $\hbox {N}^*$ conditioning on $\hbox {A}^*=0.999$), which may make model estimates unstable due to near-collinearity. Therefore, we decided to log-transform the raw measurements, and then standardize them to have mean zero and equal variance. So we obtained $A = scale(log(A^*))$. To eliminate collinearity, we decided to analyze the mean and the differences of the scaled and transformed T and N measures. Therefore, our mean measure, ‘M’, is defined as $M=(scale(\log T^*)+scale(\log N^*))/2$ and our difference measure, ‘D’ is $D=scale(\log N^*)-scale(\log T^*)$, where the *scale* function does both centering and scaling to have variance 1. This reduces the correlation between M and D to nearly zero, yet retains moderate correlations between the new measures and A. We will refer to the standardized and transformed phenotypes as A/M/D.

### Gene clusters

In order to strike a balance between computational feasibility and the desire to account for correlations between probes, we grouped together the methylation data from probes in and near each gene to form clusters of probes termed ‘gene clusters’. Each gene cluster includes probes in the gene body or within flanking regions of +/- 100 kb^63,64^ based on the manifest file of the Illumina Infinium MethylationEPIC Beadchip annotated to genome assembly hg38^54^, and these regions should capture many of the nearby regulatory sites. Gene clusters containing more than 300 probes (1845 genes, 6.47% of all genes) were split into multiple non-overlapping, consecutive regions with at most 300 probes for computational feasibility. The total number of gene clusters after analyzed is 30,576. Most of the probes (776,848 probes; 96.75% of those that passed our quality control) were included in at least one of these gene clusters. The distribution of the size of each cluster is presented in Supplementary Fig. 3. We have also examined the relationship between the number of probes selected by our method and the size of the cluster, and we found no association between them both in simulation data and in real ADNI data (Supplementary Fig. 3).

### Statistical modelling

In order to efficiently account for the many factors that can influence methylation levels in general, and to distinguish these from factors specific to AD, we proposed a two-stage modelling strategy (see Fig. 1). In the first stage, we adjusted the methylation values for covariates likely to have widespread effects, namely age, sex, years of education as a proxy for socio-economic status, cell type proportions, and epigenetic age acceleration. Epigenetic age acceleration (*eaa*) was calculated as the differences between epigenetic age^14^ and chronological age. This difference can capture general epigenetic trends in aging that may differ from chronological age and can be associated with disease. Estimated cell type proportions were dimension-reduced using principal component analysis, and the first three components were included in the models. Specifically, let the logit-transformed methylation value for individual *i* and methylation probe *j* be denoted $M_{ij}$, let *yoe* be years of education, *age* be calendar age, and *PC*1-*PC*3 be the principal components of cell type. In stage 1, we fit linear models for each probe as follows:

1 $$\begin{aligned} \text {logit}(M_{ij}) = \beta _{0j}+\beta _{1j}*\text {yoe}_i + \beta _{2j}*\text {age}_i + \beta _{3j}*\text {eaa}_i + \beta _{4j}*\text {sex}_i + \beta _{5j}*\text {PC1}_i + \beta _{6j}*\text {PC2}_i+\beta _{7j}*\text {PC3}_i + \epsilon _{ij} \end{aligned}$$

where $\epsilon _{ij}$ denotes residual error, and $\beta _{pj}$ are the linear model coefficients for covariate *p* for probe *j*. Residuals from these models are called $X_{ij}$.

Residuals from the first stage, $X_{ij}$ were then used in our second stage models that looked for associations with CSF biomarkers. By assuming no interaction between the first stage covariates and the second stage covariates, we reduced the model dimensions in the second stage and hoped to thereby increase power to detect associations of interest with the CSF biomarkers. In this second stage, we compared two strategies for analysis: one where these three measurements were analyzed simultaneously in our multivariate model, *missonet*, and another where each CSF measure and each probe’s residuals were analyzed as a separate pair. In all second stage models, the *APOE4* allele count (*APOE4*) and the AD diagnosis (DX) were included as covariates, since both have known large effects on the CSF values^60^. The details for these two strategies are presented below.

#### Epigenome-wide association study (EWAS) analysis

First we undertook a standard EWAS, analyzing each of the CSF biomarkers separately. For each transformed biomarker (A, M, D), linear models were fit, where covariates included one probe’s standardized residual $X_{ij}$ obtained from the first stage, the *APOE4* allele count (APOE4), and AD diagnosis (DX). Specifically, for each biomarker $k \in \{ A, M, D\}$ and each probe *j*, let $Y_{ik}$ represent the CSF biomarker *k* in individual *i*. The model can be written as:

2 $$\begin{aligned} Y_{ik} = \beta _{0jk}+ \beta _{1jk}*DX_{i}+\beta _{2jk} *APOE4_{i}+\beta _{3jk} *X_{ij}+\epsilon ^*_{ijk}, \end{aligned}$$

where $\epsilon ^*_{ijk}$ represents the residual errors. Slope coefficients are again represented by $\beta$, but this time with an extra subscript *k* corresponding to the three biomarkers. Estimates of slope coefficients, $\hat{\beta }_{pjk}$, were obtained using the *lm* function.

We adjusted our EWAS test statistics by using the bacon method^38^. This correction is designed to estimate and correct for potential inflation of the distribution of test statistics under the null hypothesis, and such inflation has often been observed in EWAS studies, possibly due to slight violations of distributional assumptions or due to hidden confounding^65,66^. The bacon method estimates a three-component mixture model for a set of test statistics, and assumes the central distribution follows the null hypothesis, without requiring this central distribution to have a standard normal form. We used the function *bacon* from the package of the same name (version 1.26.0) to adjust for EWAS *p* values. These adjusted *p* values were used for the downstream analyses. We used threshold $\alpha =\frac{3.6e-8}{3}$ for genome-wide significance^39^ after correcting for multiple testing, and we used a suggestive level of statistical significance of $\alpha =\frac{1e-5}{3}$^39^.

#### L1-penalized multivariate methods

Our goal was to jointly analyse the three CSF biomarkers (residuals after stage 1), using *missoNet*^40^. Typically, a penalized multivariate regression can be presented as an optimisation problem, where the goal is to estimate the regression coefficients and the inverse covariance matrix of the residuals so that the model fits the data well while satisfying certain regularisation constraints to control overfitting and ensure stability of the estimates. The optimization problem can be formulated as:

3 $$\begin{aligned} \underset{\varvec{\Gamma }, \varvec{\Theta } \succeq 0}{\text {min}}\ \textrm{tr}[(\varvec{Y} - \varvec{X\Gamma })^\top (\varvec{Y}-\varvec{X\Gamma }) \varvec{\Theta }] -\log (\det (\varvec{\Theta })) +\lambda \text {P}(\varvec{\Gamma }) +\rho \text {Q}(\varvec{\Theta }) \end{aligned}$$

where $\varvec{Y}$ represents a matrix of size $n\times q$ of *q* response variables, $\varvec{X}$ is the $n\times p$ matrix of predictor variables, $\varvec{\Gamma }$ (size $p \times q$) is the matrix of regression coefficients and $\varvec{\Theta }$ is the inverse covariance matrix of the residuals (size $q \times q$). We use $\text {P}(\cdot )$ and $\text {Q}(\cdot )$ to represent L1 penalty functions applied to the regression coefficients and the inverse covariance, helping to achieve a more robust and interpretable model. The terms $\lambda$ and $\rho$ are tuning parameters that control the strength of the regularisation on $\varvec{\Gamma }$ and $\varvec{\Theta }$, respectively.

In order to strike a balance between computational feasibility and the desire to account for correlations between probes, we analyzed gene-based clusters of probes simultaneously (see section *Gene clusters* above). Therefore, our second-stage models were constructed as follows:

4 $$\begin{aligned} \varvec{Y_i}&=\begin{bmatrix} A_i \\ M_i \\ D_i \end{bmatrix} \nonumber \\ \log (\varvec{Y_i})&=\varvec{\gamma _{0j}}+\varvec{\gamma _{1j}} *DX_i+\varvec{\gamma _{2j}} *APOE4_{i}+\varvec{\Gamma _{3j}}*\varvec{X_{ij}}+\varvec{\epsilon _{ij}}. \end{aligned}$$

Here, as before, $DX_i$ represents the AD diagnosis and $APOE4_i$ represents the *APOE4* allele count for individual *i*. Their corresponding slope coefficients $\varvec{(\gamma _{0j},\gamma _{1j},\gamma _{2j})}$ are vectors of size 3 for the three phenotypes. The slope coefficient for the probe residuals $\varvec{X_{ij}}$ (size $m*1$), represented by $\varvec{\Gamma _{3j}}$, is a matrix of size $3*m$ that corresponds to all *m* probes in the gene cluster *j* as well as three coefficients for A, M, and D. The vector of three residuals for cluster *j* is denoted by $\varvec{\epsilon _{ij}}$.

For the *missoNet* package (Version 1.2.0), we used BIC as implemented within the *missoNet* function choosing *GoF=‘BIC’* and chose the penalization values of $\lambda$ and $\rho$ that minimized the corresponding BIC. Our final models then contain only the probes in each cluster estimated to have at least one non-zero slope coefficient in $\varvec{\gamma _{3j}}$ for the relationships between a biomarker and the methylation in cluster *j*.

### Simulation study

We conducted simulations to evaluate both type 1 error and statistical power, using datasets with and without predefined associations. The model used in the simulation can be found in section *Statistical modelling*.

#### Simulated datasets

First, we selected 50 gene clusters at random from the ADNI methylation dataset to reflect realistic correlation structures. The methylation data utilized in the simulations comprised residuals from the stage 1 analysis described in Section *Statistical modelling*. Each simulated dataset included true values for AD diagnosis status (*DX*) and *APOE4* allele counts (*APOE*4). Effect sizes for these two covariates were randomly assigned to each of the three biomarker phenotypes, drawn from uniform distributions Unif(0.1,0.5) or Unif(-0.5,-0.1). The magnitude of these effect sizes was informed by previously observed univariate EWAS results, where the estimated values lay in these ranges. We denote these assigned parameters as ${\beta _{dx}} \in \mathbb {R}^{3 \times 1}$ and ${\beta _{apoe4}} \in \mathbb {R}^{3 \times 1}$. The three phenotypes of interest, $Y^*$, were then generated using:

$$\begin{aligned} Y^*={\beta _{dx}}*DX+{\beta _{apoe4}}*APOE4+\mathbf {\beta _p}*X^*+e^*. \end{aligned}$$

Here, the random error term $e^*$ was i.i.d. from a multivariate normal distribution MVN(0,$\Sigma ^2$), where $\Sigma ^*$ was the empirical phenotype variance-covariance matrix from the transformed A/M/D values. The coefficients $\mathbf {\beta _p}$ (dimension $3 \times m$, with *m* representing cluster size) varied according to simulation conditions and the intended strength of associations. The values were also informed by the estimated effect sizes ($\hat{\mathbf {\beta _p}}$) from prior EWAS analyses: 0 for the null model, 0.04 for median observed effect size, and 0.08 for the highest observed effect size.

#### Parameter settings

We have defined the following simulation scenarios for the parameter matrix $\mathbf {\beta _p}$:

1. **Null model**: No probes are associated with any phenotype, hence $\mathbf {\beta _p} = \textbf{0}$.
2. **Moderate signals across three phenotypes**: Within each cluster, we randomly selected 5 matrix elements with moderate effect sizes (0.04). These signals were evenly distributed across the three phenotypes, resulting in approximately 5/3 associated probes per phenotype.
3. **Strong signals across three phenotypes**: Similar to the moderate scenario, but effect sizes were stronger (0.08). Again, 5 elements per cluster were randomly selected and evenly assigned across the three phenotypes.

Additionally, we explored a scenario with moderate signals on only one phenotype. In this setting, we randomly selected 5 probes within each cluster and assigned them a moderate effect size (0.04), but exclusively for one phenotype. This specific scenario was introduced to assess the impact of *cglasso*’s additional penalization, which is sensitive to phenotype variance, comparing with *missoNet* that does not include this extra penalization. While this extra penalization may enhance computational stability, it potentially introduces greater selection bias. We deliberately chose not to center and scale the phenotypes again, prior to fitting, to create conditions where *missonet* and *cglasso* will penalize differently. Detailed discussions and results for this scenario are provided in Supplementary Section 2.

#### Performance evaluation

We assessed the performance for each set of simulations across the 50 simulated datasets using three metrics: (1) **True Positive Rate (TPR)**: Defined as the ratio of correctly identified signals (true positives) to the total number of truly associated signals. 2) **True Negative Rate (TNR)**: Calculated as the ratio of correctly identified non-associated signals (true negatives) to the total number of truly non-associated signals. 3) **Matthews Correlation Coefficient (MCC)**: This metric evaluates the balance between true positives (TP), true negatives (TN), false positives (FP), and false negatives (FN). It is computed as:

$$\begin{aligned} MCC=\frac{TP*TN-FP*FN}{\sqrt{(TP+FP)(TP+FN)(TN+FP)(TN+FN)}} \end{aligned}$$

where *FP=*$1-$*TN* and *FN=*$1-$*TP*. We selected the MCC as the primary performance measure because it simultaneously accounts for true positives and true negatives, making it particularly suitable for evaluating performance in sparse scenarios, such as those encountered in our analyses.

## Supplementary Information

Supplementary Information 1.

Supplementary Information 2.

## Data Availability

The DNA methylation data and CSF biomarker datasets were from the ADNI which can be accessed from http://adni.loni.usc.edu. The replication study used CLSA data: Data are available from the Canadian Longitudinal Study on Aging (www.clsa-elcv.ca) for researchers who meet the criteria for access to de-identified CLSA data. The source codes for data analyses performed in this study can be accessed from the github page: https://github.com/GreenwoodLab/ADNI_code. The full EWAS results can also be found at the same github page.
