# Supplementary material for: A multivariate approach to identify association between peripheral blood DNA methylation and cerebrospinal fluid biomarkers of Alzheimer disease
Source: Sci Rep. 2025 Nov 3;15:38289. doi: 10.1038/s41598-025-22004-3 (PMC12583527; doi:10.1038/s41598-025-22004-3)
Supplement: Supplementary file 1 — Supplementary Information 1. [file 41598_2025_22004_MOESM1_ESM.pdf]

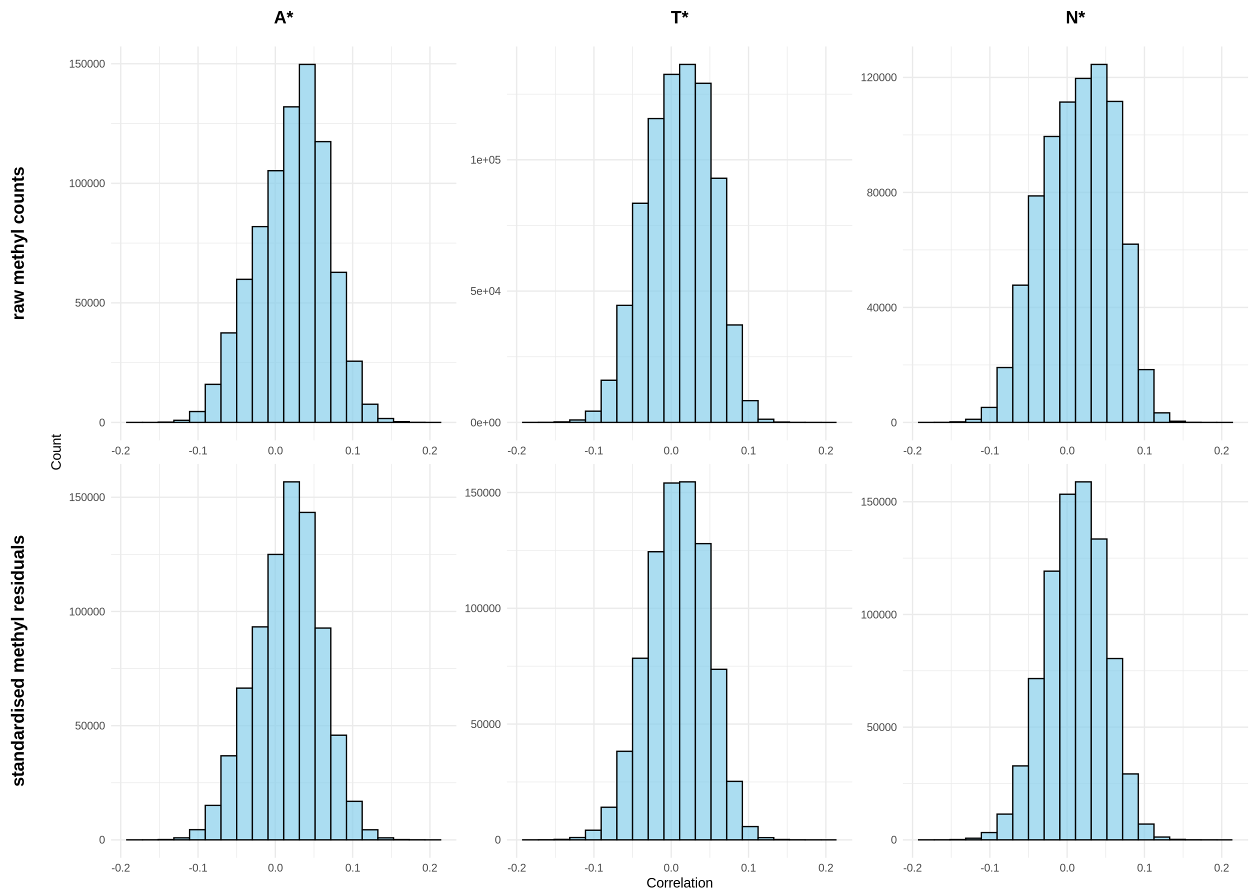

Supplementary Figure 1: Correlations between individual DNA methylation probes and the 3 original CSF biomarkers (A*,T*,N*). For each CSF biomarker, correlations were estimated for both the raw methylation measurements (row 'Raw methylation counts') and the standardized methylation residuals obtained from stage 1 (row 'Standardised methylation residuals'). Most of the methylation probes had small correlations with the CSF biomarkers. Some probes showed correlations as large as 0.2.

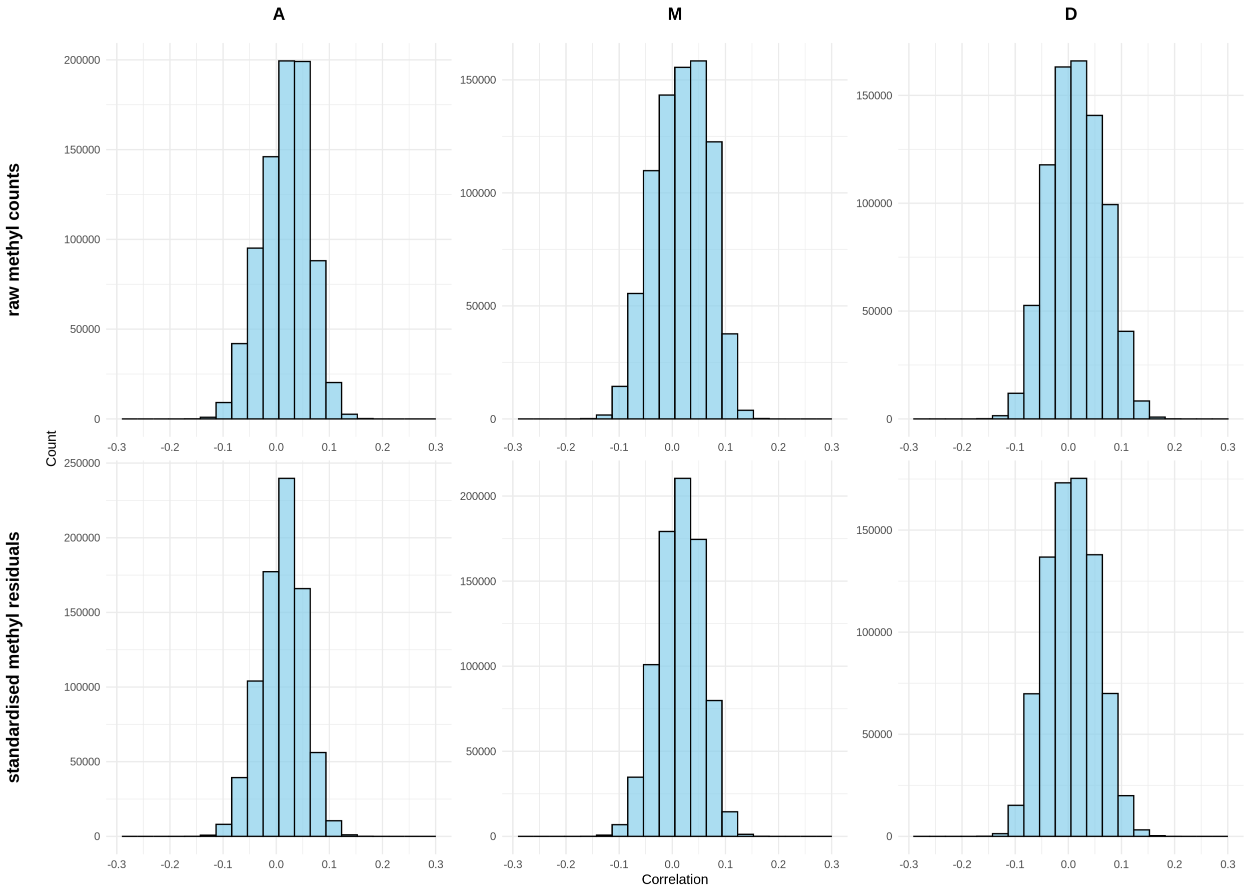

Supplementary Figure 2: Correlations between individual DNA methylation probes and the transformed CSF biomarkers (A/M/D). For each CSF biomarker, correlations were estimated for both the raw methylation measurements (top row) and the standardised methylation residuals obtained from stage 1 (bottom row). Most of the methylation probes had small correlations with the CSF biomarkers. The highest correlations from both raw methylation and standardized residuals are around ± 0.3

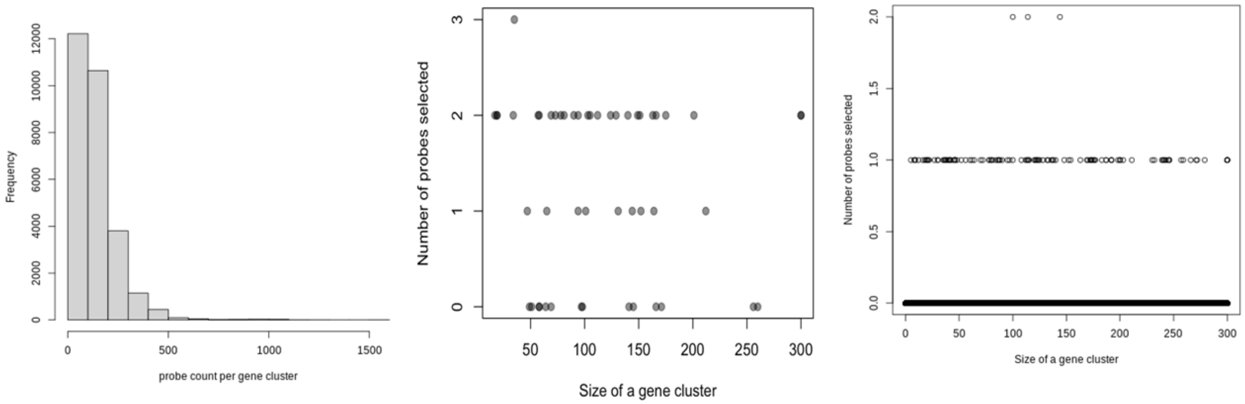

Supplementary Figure 3: **Left:** Distribution of number of probes per gene cluster across the genome, before dividing gene clusters larger than 300 probes into smaller sets. **Middle:** Number of probes selected by *missoNet*-BIC in a gene cluster with respect to cluster size calculated from simulated dataset. **Right:** Number of probes selected by *missoNet*-BIC in a gene cluster with respect to cluster size calculated from the ADNI data. There is no association shown here between the size of a gene cluster and how many probes are selected in a gene cluster both in simulated data and in the ADNI data.

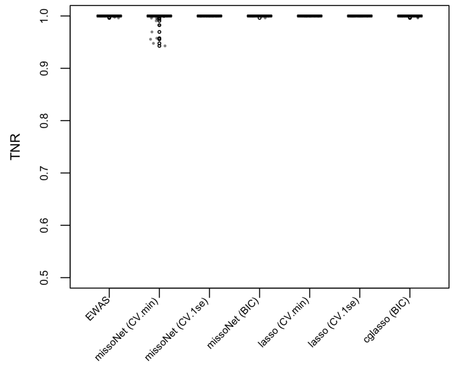

Supplementary Figure 4: True negative rate (TNR) for each method in simulation when no true associations are present. The results for each simulated dataset (grey dots) are overlaid with boxplots.

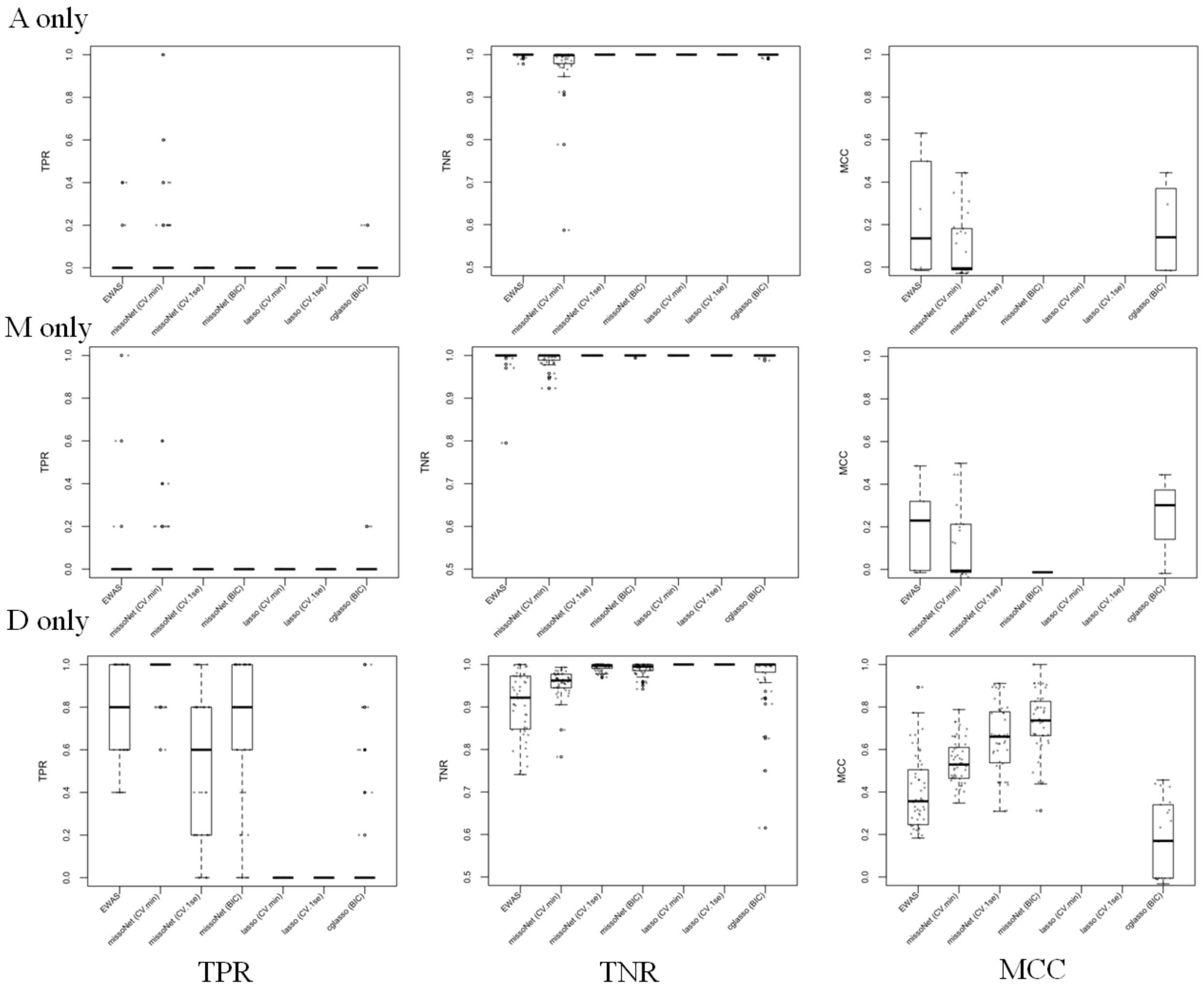

Supplementary Figure 5: Performance of different methods in simulations when the true associations (effect size =0.04) affect only one phenotype. Top row shows results when associations are solely with *A*, middle row shows associations solely with *M*, and bottom row shows associations solely with *D*. The results for each simulated dataset (grey dots) are overlaid with boxplots. *TPR* True positive rate; *TNR* True negative rate; *MCC* Matthews correlation coefficient.

Supplementary Section 1: Replication study using data from the Canadian Longitudinal Study on Aging

We used data from the Canadian Longitudinal Study on Aging (CLSA) (project #2104015) to replicate our findings in ADNI. We obtained their demographic data as well as methylation data on 1478 individuals. In the CLSA dataset, there is information on self-reported AD status, but no clinical diagnosis. We also analyzed a cognitive test, REYII, as a second phenotype related to AD. We are lacking the genotypes for the *APOE4*, and there were no CSF biomarkers measured in CLSA. Therefore, we tested associations for the 34 probes in Table 2 using the formula below:

$$g(AD/REYII) = \beta_{0j} + \beta_{1j} * \text{yoe}_i + \beta_{2j} * \text{age}_i + \beta_{3j} * \text{eaa}_i + \beta_{4j} * \text{sex}_i + \beta_{5j} * \text{PC1}_i + \beta_{6j} * \text{PC2}_i + \beta_{7j} * \text{PC3}_i + \beta_{8j} * \text{probe} + \varepsilon_{ij},$$

where "AD/REYII" implies that we fit models for each of these phenotypes separately. The two phenotypes were modelled using generalized linear models with different link functions. For reported AD, we used the *logit* function, and for the cognitive test REYII, we used a *quasi-poisson* function since this is count data. We summarized the replication results in Supplementary Tables 5 and 6.

Supplementary Section 2: Simulation Results

As mentioned in the Methods section, we conducted simulations for several different scenarios. This section shows additional scenarios that were not in the Results section. First, we present here the performance of each of the methods when no true association exists (i.e. a null model). Since there are no true associations, the true positive rates will all be zero, and MCC is not computable. Thus, we are only showing the true negative rate results in Supplementary Figure 4. All methods perform similarly.

In Supplementary Figure 5 we show three simulation scenarios where all true signals lie in just one phenotype: A, M, or D. These scenarios represents different detection powers given the same fixed true effect size: A and M both have low detection power, and the performances of all methods are more vulnerable to 2-3 dataset that select the true associated probes by random. On the other hand, D has a higher detection power, and that most of the methods can reliably and robustly identify true signals (see TPR performance from Supplementary Figure 5). In this case, *missoNet*-BIC achieves the most balanced performance (the highest MCC), reflecting its superior ability to differentiate true associations from noises.

The difference we see in detection power among these phenotypes is due to their variance differences. Given a fixed sample size, phenotypes with larger variances result in a lower detection power for a given effect size. Since both A and M have higher variance, all methods have very low detection power when true signals lies on these two phenotypes. On the other hand, D is inherently less variable than A and M, and thus the same effect size yields a higher signal-to-noise ratio, resulting a substantial increase of the detection power and MCC for most methods.

Supplementary Section 3: Interaction models

We are also interested in understanding whether an interaction may exist between DNA methylation probes and our AD-specific covariates (namely, AD diagnosis and *APOE4* count). We were not able to use a similar multivariate approach as in our main analyses to answer this question for the following reasons. Firstly, the number of covariates in the second stage model could be very large, since the interactions would need to include all probes in an extended gene region. Secondly, *missoNet*, as implemented, cannot guarantee an appropriate inclusion of a hierarchy of terms in an interaction (i.e. to keep the two main effects when an interaction is retained). Such hierarchies are crucial to interpretability. Instead, we have conducted single-phenotype analyses on the three phenotypes separately for the 34 probes identified by *missoNet* in Table 2, and investigated two-way interactions with AD diagnosis and *APOE4*. Namely, we fit

$$Y = \beta_0 + \beta_1 * \text{probe} + \beta_2 * \text{AD} + \beta_3 * \text{APOE4} + \beta_{12} * \text{probe} * \text{AD} + \beta_{13} * \text{probe} * \text{APOE4}$$

where *probe* refers to the methylation residuals obtained from stage 1.
The summary statistics for the two interaction terms are summarized in Supplementary Tables 7-9.

Supplementary Section 4: ADNI authors' list

The investigators within the ADNI contributed to the design and implementation of ADNI and/or provided data are provided here, but they do not participate in analysis or writing of this report. The full list can also be found using the following link: https://adni.loni.usc.edu/wp-content/themes/freshnews-dev-v2/documents/policy/ADNI%20Groups%20Acknowledgements%20Journal%20Format.pdf

Michael Weiner, MD (UC San Francisco, Principal Investigator, Executive Committee); Paul Aisen, MD (UC San Diego, ADCS PI and Director of Coordinating Center Clinical Core, Executive Committee, Clinical Core Leaders); Ronald Petersen, MD, PhD (Mayo Clinic, Rochester, Executive Committee, Clinical Core Leader); Clifford R. Jack, Jr., MD (Mayo Clinic, Rochester, Executive Committee, MRI Core Leader); William Jagust, MD (UC Berkeley, Executive Committee; PET Core Leader); John Q. Trojanowki, MD, PhD (U Pennsylvania, Executive Committee, Biomarkers Core Leader); Arthur W. Toga, PhD (USC, Executive Committee, Informatics Core Leader); Laurel Beckett, PhD (UC Davis, Executive Committee, Biostatistics Core Leader); Robert C. Green, MD, MPH (Brigham and Women's Hospital, Harvard Medical School, Executive Committee and Chair of Data and Publication Committee); Andrew J. Saykin, PsyD (Indiana University, Executive Committee, Genetics Core Leader); John Morris, MD (Washington University St. Louis, Executive Committee, Neuropathology Core Leader); Leslie M. Shaw (University of Pennsylvania, Executive Committee, Biomarkers Core Leader); Enchi Liu, PhD (Janssen Alzheimer Immunotherapy, ADNI 2 Private Partner Scientific Board Chair); Tom Montine, MD, PhD (University of Washington) ; Ronald G. Thomas, PhD (UC San Diego); Michael Donohue, PhD (UC San Diego); Sarah Walter, MSc (UC San Diego); Devon Gessert (UC San Diego); Tamie Sather, MS (UC San Diego,); Gus Jiminez, MBS (UC San Diego); Danielle Harvey, PhD (UC Davis;); Michael Donohue, PhD (UC San Diego); Matthew Bernstein, PhD (Mayo Clinic, Rochester); Nick Fox, MD (University of London); Paul Thompson, PhD (USC School of Medicine); Norbert Schuff, PhD (UCSF MRI); Charles DeCArli, MD (UC Davis); Bret Borowski, RT (Mayo Clinic); Jeff Gunter, PhD (Mayo Clinic); Matt Senjem, MS (Mayo Clinic); Prashanthi Vemuri, PhD (Mayo Clinic); David Jones, MD (Mayo Clinic); Kejal Kantarci (Mayo Clinic); Chad Ward (Mayo Clinic); Robert A. Koeppe, PhD (University of Michigan, PET Core Leader); Norm Foster, MD (University of Utah); Eric M. Reiman, MD (Banner Alzheimer's Institute); Kewei Chen, PhD (Banner Alzheimer's Institute); Chet Mathis, MD (University of Pittsburgh); Susan Landau, PhD (UC Berkeley); Nigel J. Cairns, PhD, MRCPath (Washington University St. Louis); Erin Householder (Washington University St. Louis); Lisa Taylor Reinwald, BA, HTL (Washington University St. Louis); Virginia Lee, PhD, MBA (UPenn School of Medicine); Magdalena Korecka, PhD (UPenn School of Medicine); Michal Figurski, PhD (UPenn School of Medicine); Karen Crawford (USC); Scott Neu, PhD (USC); Tatiana M. Foroud, PhD (Indiana University); Steven Potkin, MD UC (UC Irvine); Li Shen, PhD (Indiana University); Faber Kelley, MS, CCRC (Indiana University); Sungeun Kim, PhD (Indiana University); Kwangsik Nho, PhD (Indiana University); Zaven Kachaturian, PhD (Khachaturian, Radebaugh & Associates, Inc and Alzheimer's Association's Ronald and Nancy Reagan's Research Institute); Richard Frank, MD, PhD (General Electric); Peter J. Snyder, PhD (Brown University); Susan Molchan, PhD (National Institute on Aging/ National Institutes of Health); Jeffrey Kaye, MD (Oregon Health and Science University); Joseph Quinn, MD (Oregon Health and Science University); Betty Lind, BS (Oregon Health and Science University); Raina Carter, BA (Oregon Health and Science University); Sara Dolen, BS (Oregon Health and Science University); Lon S. Schneider, MD (University of Southern CaliforGroups Acknowledgements Journal Format nia); Sonia Pawluczyk, MD (University of Southern California); Mauricio Beccera, BS (University of Southern California); Liberty Teodoro, RN (University of Southern California); Bryan M. Spann, DO, PhD (University of Southern California); James Brewer, MD, PhD (University of California San Diego); Helen Vanderswag, RN (University of California San Diego); Adam Fleisher, MD (University of California San Diego); Judith L. Heidebrink, MD, MS (University of Michigan); Joanne L. Lord, LPN, BA, CCRC (University of Michigan); Ronald Petersen, MD, PhD (Mayo Clinic, Rochester); Sara S. Mason, RN (Mayo Clinic, Rochester); Colleen S. Albers, RN (Mayo Clinic, Rochester); David Knopman, MD (Mayo Clinic, Rochester); Kris Johnson, RN (Mayo Clinic, Rochester); Rachelle S. Doody, MD, PhD (Baylor College of Medicine); Javier Villanueva Meyer, MD (Baylor College of Medicine); Munir Chowdhury, MBBS, MS (Baylor College of Medicine); Susan Rountree, MD (Baylor College of Medicine); Mimi Dang, MD (Baylor College of Medicine); Yaakov Stern, PhD (Columbia University Medical Center); Lawrence S. Honig, MD, PhD (Columbia University Medical Center); Karen L. Bell, MD (Columbia University Medical Center); Beau Ances, MD (Washington University, St. Louis); John C. Morris, MD (Washington University, St. Louis); Maria Carroll, RN, MSN (Washington University, St. Louis); Sue Leon, RN, MSN (Washington University, St. Louis); Erin Householder, MS, CCRP (Washington University, St. Louis); Mark A. Mintun, MD (Washington University, St. Louis); Stacy Schneider, APRN, BC, GNP (Washington University, St. Louis); Angela Oliver, RN, BSN, MSG ; Daniel Marson, JD, PhD (University of Alabama Birmingham); Randall Griffith, PhD, ABPP (University of Alabama Birmingham); David Clark, MD (University of Alabama Birmingham); David Geldmacher, MD (University of Alabama Birmingham); John Brockington, MD (University of Alabama Birmingham); Erik Roberson, MD (University of Alabama Birmingham); Hillel Grossman, MD (Mount Sinai School of Medicine); Effie Mitsis, PhD (Mount Sinai School of Medicine); Leyla deToledo-Morrell, PhD (Rush University Medical Center); Raj C. Shah, MD (Rush University

Medical Center); Ranjan Duara, MD (Wien Center); Daniel Varon, MD (Wien Center); Maria T. Greig, HP (Wien Center); Peggy Roberts, CNA (Wien Center); Marilyn Albert, PhD (Johns Hopkins University); Chiadi Onyike, MD (Johns Hopkins University); Daniel D'Agostino II, BS (Johns Hopkins University); Stephanie Kielb, BS (Johns Hopkins University); James E. Galvin, MD, MPH (New York University); Dana M. Pogorelec (New York University); Brittany Cerbone (New York University); Christina A. Michel (New York University); Henry Rusinek, PhD (New York University); Mony J de Leon, EdD (New York University); Lidia Glodzik, MD, PhD (New York University); Susan De Santi, PhD (New York University); P. Murali Doraiswamy, MD (Duke University Medical Center); Jeffrey R. Petrella, MD (Duke University Medical Center); Terence Z. Wong, MD (Duke University Medical Center); Steven E. Arnold, MD (University of Pennsylvania); Jason H. Karlawish, MD (University of Pennsylvania); David Wolk, MD (University of Pennsylvania); Charles D. Smith, MD (University of Kentucky); Greg Jicha, MD (University of Kentucky); Peter Hardy, PhD (University of Kentucky); Partha Sinha, PhD (University of Kentucky); Elizabeth Oates, MD (University of Kentucky); Gary Conrad, MD (University of Kentucky); Oscar L. Lopez, MD (University of Pittsburgh); MaryAnn Oakley, MA (University of Pittsburgh); Donna M. Simpson, CRNP, MPH (University of Pittsburgh); Anton P. Porsteinsson, MD (University of Rochester Medical Center); Bonnie S. Goldstein, MS, NP (University of Rochester Medical Center); Kim Martin, RN (University of Rochester Medical Center); Kelly M. Makino, BS (University of Rochester Medical Center); M. Saleem Ismail, MD (University of Rochester Medical Center); Connie Brand, RN (University of Rochester Medical Center); Ruth A. Mulnard, DNSc, RN, FAAN (University of California, Irvine); Gaby Thai, MD (University of California, Irvine); Catherine Mc Adams Ortiz, MSN, RN, A/GNP (University of California, Irvine); Kyle Womack, MD (University of Texas Southwestern Medical School); Dana Mathews, MD, PhD (University of Texas Southwestern Medical School); Mary Quiceno, MD (University of Texas Southwestern Medical School); Ramon Diaz Arrastia, MD, PhD (University of Texas Southwestern Medical School); Richard King, MD (University of Texas Southwestern Medical School); Myron Weiner, MD (University of Texas Southwestern Medical School); Kristen Martin Cook, MA (University of Texas Southwestern Medical School); Michael DeVous, PhD (University of Texas Southwestern Medical School); Allan I. Levey, MD, PhD (Emory University); James J. Lah, MD, PhD (Emory University); Janet S. Cellar, DNP, PMHCNS BC (Emory University); Jeffrey M. Burns, MD (University of Kansas, Medical Center); Heather S. Anderson, MD (University of Kansas, Medical Center); Russell H. Swerdlow, MD (University of Kansas, Medical Center); Liana Apostolova, MD (University of California, Los Angeles); Kathleen Tingus, PhD (University of California, Los Angeles); Ellen Woo, PhD (University of California, Los Angeles); Daniel H.S. Silverman, MD, PhD (University of California, Los Angeles); Po H. Lu, PsyD (University of California, Los Angeles); George Bartzokis, MD (University of California, Los Angeles); Neill R Graff Radford, MBBCH, FRCP (London) (Mayo Clinic, JacksonGroups ville); Francine Parfitt, MSH, CCRC (Mayo Clinic, Jacksonville); Tracy Kendall, BA, CCRP (Mayo Clinic, Jacksonville); Heather Johnson, MLS, CCRP (Mayo Clinic, Jacksonville); Martin R. Farlow, MD (Indiana University); Ann Marie Hake, MD (Indiana University); Brandy R. Matthews, MD (Indiana University); Scott Herring, RN, CCRC (Indiana University); Cynthia Hunt, BS, CCRP (Indiana University); Christopher H. van Dyck, MD (Yale University School of Medicine); Richard E. Carson, PhD (Yale University School of Medicine); Martha G. MacAvoy, PhD (Yale University School of Medicine); Howard Chertkow, MD (McGill Univ., Montreal Jewish General Hospital); Howard Bergman, MD (McGill Univ., Montreal Jewish General Hospital); Chris Hosein, Med (McGill Univ., Montreal Jewish General Hospital); Sandra Black, MD, FRCPC (Sunnybrook Health Sciences, Ontario); Dr Bojana Stefanovic (Sunnybrook Health Sciences, Ontario); Curtis Caldwell, PhD (Sunnybrook Health Sciences, Ontario); Ging Yuek Robin Hsiung, MD, MHSc, FRCPC (U.B.C. Clinic for AD & Related Disorders); Howard Feldman, MD, FRCPC (U.B.C. Clinic for AD & Related Disorders); Benita Mudge, BS (U.B.C. Clinic for AD & Related Disorders); Michele Assaly, MA Past (U.B.C. Clinic for AD & Related Disorders); Andrew Kertesz, MD (Cognitive Neurology St. Joseph's, Ontario); John Rogers, MD (Cognitive Neurology St. Joseph's, Ontario); Dick Trost, PhD (Cognitive Neurology St. Joseph's, Ontario); Charles Bernick, MD (Cleveland Clinic Lou Ruvo Center for Brain Health); Donna Munic, PhD (Cleveland Clinic Lou Ruvo Center for Brain Health); Diana Kerwin, MD (Northwestern University); Marek Marsel Mesulam, MD (Northwestern University); Kristine Lipowski, BA (Northwestern University); Chuang Kuo Wu, MD, PhD (Northwestern University); Nancy Johnson, PhD (Northwestern University); Carl Sadowsky, MD (Premiere Research Inst (Palm Beach Neurology)); Walter Martinez, MD (Premiere Research Inst (Palm Beach Neurology)); Teresa Villena, MD (Premiere Research Inst (Palm Beach Neurology)); Raymond Scott Turner, MD, PhD (Georgetown University Medical Center); Kathleen Johnson, NP (Georgetown University Medical Center); Brigid Reynolds, NP (Georgetown University Medical Center); Reisa A. Sperling, MD (Brigham and Women's Hospital); Keith A. Johnson, MD (Brigham and Women's Hospital); Gad Marshall, MD (Brigham and Women's Hospital); Meghan Frey (Brigham and Women's Hospital); Jerome Yesavage, MD (Stanford University); Joy L. Taylor, PhD (Stanford University); Barton Lane, MD (Stanford University); Allyson Rosen, PhD (Stanford University); Jared Tinklenberg, MD (Stanford University); Marwan N. Sabbagh, MD (Banner Sun Health Research Institute); Christine M. Belden, PsyD (Banner Sun Health Research Institute); Sandra A. Jacobson, MD (Banner Sun Health Research Institute); Sherye A. Sirrel, MS (Banner Sun Health Research Institute); Neil Kowall, MD (Boston University); Ronald Killiany, PhD (Boston University); Andrew E. Budson, MD (Boston University); Alexander Norbash, MD (Boston University); Patricia Lynn Johnson, BA (Boston University); Thomas O. Obisesan, MD,

MPH (Howard University); Saba Wolday, MSc (Howard University); Joanne Allard, PhD (Howard University); Alan Lerner, MD (Case Western Reserve University); Paula Ogrocki, PhD (Case Western Reserve University); Leon Hudson, MPH (Case Western Reserve University); Evan Fletcher, PhD (University of California, Davis Sacramento); Owen Carmichael, PhD (University of California, Davis Sacramento); John Olichney, MD (University of California, Davis Sacramento); Charles DeCarli, MD (University of California, Davis Sacramento); Smita Kittur, MD (Neurological Care of CNY); Michael Borrie, MB ChB (Parkwood Hospital); T Y Lee, PhD (Parkwood Hospital); Dr Rob Bartha, PhD (Parkwood Hospital); Sterling Johnson, PhD (University of Wisconsin); Sanjay Asthana, MD (University of Wisconsin); Cynthia M. Carlsson, MD (University of Wisconsin); Steven G. Potkin, MD (University of California, Irvine BIC); Adrian Preda, MD (University of California, Irvine BIC); Dana Nguyen, PhD (University of California, Irvine BIC); Pierre Tariot, MD (Banner Alzheimer's Institute); Adam Fleisher, MD (Banner Alzheimer's Institute); Stephanie Reeder, BA (Banner Alzheimer's Institute); Vernice Bates, MD (Dent Neurologic Institute); Horacio Capote, MD (Dent Neurologic Institute); Michelle Rainka, PharmD, CCRP (Dent Neurologic Institute); Douglas W. Scharre, MD (Ohio State University); Maria Kataki, MD, PhD (Ohio State University); Anahita Adeli, MD (Ohio State University); Earl A. Zimmerman, MD (Albany Medical College); Dzintra Celmins, MD (Albany Medical College); Alice D. Brown, FNP (Albany Medical College); Godfrey D. Pearlson, MD (Hartford Hosp, Olin Neuropsychiatry Research Center); Karen Blank, MD (Hartford Hosp, Olin Neuropsychiatry Research Center); Karen Anderson, RN (Hartford Hosp, Olin Neuropsychiatry Research Center); Robert B. Santulli, MD (Dartmouth Hitchcock Medical Center); Tamar J. Kitzmiller (Dartmouth Hitchcock Medical Center); Eben S. Schwartz, PhD (Dartmouth Hitchcock Medical Center); Kaycee M. Sink, MD, MAS (Wake Forest University Health Sciences); Jeff D. Williamson, MD, MHS (Wake Forest University Health Sciences); Pradeep Garg, PhD (Wake Forest University Health Sciences); Franklin Watkins, MD (Wake Forest University Health Sciences); Brian R. Ott, MD (Rhode Island Hospital); Henry Querfurth, MD (Rhode Island Hospital); Geoffrey Tremont, PhD (Rhode Island Groups Hospital); Stephen Salloway, MD, MS (Butler Hospital); Paul Malloy, PhD (Butler Hospital); Stephen Correia, PhD (Butler Hospital); Howard J. Rosen, MD (UC San Francisco); Bruce L. Miller, MD (UC San Francisco); Jacobo Mintzer, MD, MBA (Medical University South Carolina); Kenneth Spicer, MD, PhD (Medical University South Carolina); David Bachman, MD (Medical University South Carolina); Elizabether Finger, MD (St. Joseph's Health Care); Stephen Pasternak, MD (St. Joseph's Health Care); Irina Rachinsky, MD (St. Joseph's Health Care); John Rogers, MD (St. Joseph's Health Care); Andrew Kertesz, MD (St. Joseph's Health Care); Dick Drost, MD (St. Joseph's Health Care); Nunzio Pomara, MD (Nathan Kline Institute); Raymundo Hernando, MD (Nathan Kline Institute); Antero Sarrael, MD (Nathan Kline Institute); Susan K. Schultz, MD (University of Iowa College of Medicine, Iowa City); Laura L. Boles Ponto, PhD (University of Iowa College of Medicine, Iowa City); Hyungsub Shim, MD (University of Iowa College of Medicine, Iowa City); Karen Elizabeth Smith, RN (University of Iowa College of Medicine, Iowa City); Norman Relkin, MD, PhD (Cornell University); Gloria Chaing, MD (Cornell University); Lisa Raudin, PhD (Cornell University); Amanda Smith, MD (University of South Floriday: USF Health Byrd Alzheimer's Institute); Kristin Fargher, MD (University of South Floriday: USF Health Byrd Alzheimer's Institute); Balebail Ashok Raj, MD (University of South Floriday: USF Health Byrd Alzheimer's Institute)
